# Does cholesterol act as a protector of cholinergic projections in Alzheimer's disease?

**DOI:** 10.1186/1476-511X-4-13

**Published:** 2005-06-10

**Authors:** Iwo J Bohr

**Affiliations:** 1 University of Newcastle, Department of Neurology, Neurobiology and Psychiatry, Institute for Ageing and Health, Newcastle General Hospital, Westgate Road, Newcastle-upon-Tyne, NE4 6BE, UK

## Abstract

The relationship between Alzheimer's disease (AD) and progressive degeneration of the forebrain cholinergic system is very well established, whereas mechanisms linking this disease with cholesterol, apolipoprotein E (apoE) phenotype, and amyloid precursor protein (APP) metabolism have not been fully elucidated even though there is a plethora of publications separately on each of these issues. The intention of this hypothesis is to unify knowledge coming from all of these areas. It is based on an assumption that the process of APP hypermetabolism is a neuroprotective response for age-related cholinergic deterioration. In some individuals this initially positive process becomes highly overregulated by genetic or/and epigenetic risk factors and after many years of accumulations lead eventually to AD. I hypothesise that neuroprotective role of APP-hypermetabolism might be related to enrichment of neuronal membranes (lipid rafts in particular) in cholesterol in order to compensate for decrease in presynaptic cholinergic transmission and/or AD-related decrease in cholesterol levels. The above is consistent with findings indicating that activity of both muscarinic and nicotinic cholinergic receptors is correlated in a positive manner with cholesterol plasmalemmal content. Briefly – APP metabolism together with transport of cholesterol in apoE containing lipoproteins seem to play a key role in mobilising cholesterol into neuronal membranes.

## Background

The role of cholesterol in Alzheimer's disease (AD) is attracting increasing attention of researchers [1] and there are conflicting messages coming form a great deal of reports. Despite the fact that a wide-spread opinion about high levels of this lipid in the organism still remains negative, there is a growing body of evidence suggesting its beneficial role in the brain. It is for example corroborated by the study showing that high cholesterol blood levels correlate with a lower mortality index and a better outcome following a first stroke [2]. There was also a positive relationship between a hypercholesterolemic diet and improved preservation of cognitive functions in rats which previously underwent anoxic period [3]. The significance of the results obtained with the use of a dietetic paradigm has recently been confirmed by findings reporting a net flux of peripheral cholesterol through Blood-Brain Barrier in the form of 27-hydroxycholesterol [4]. It has also been found that patients suffering from AD have lower levels of cholesterol in cerebrospinal fluid[5] in the lipid fraction of brain membranes resulting in altered membrane physical properties [6] and recently in cholesterol-enriched lipid microdomains in plasmalemma – lipid rafts [7]. Moreover a relationship between AD and down-regulation of seladin-1; a protein involved in cholesterol synthesis was found, reviewed in [7] which may be due to a genetic disorder.

It appears that cholesterol has universal neuroprotective properties. However for the purpose of this article this activity will be described only in connection to AD.

The hypothesis combines within one unifying concept well established facts from the three following main streams of AD research:

- the prevalence of forebrain cholinergic system deficits in the disease development, – metabolism of amyloid precursor protein (APP) and

- the role of apolipoprotein E4 (apoE4) isoform as a risk factor in association with cholesterol metabolism in the brain.

These key issues will be briefly introduced before presenting the hypothesis.

The main type of neurons primarily affected by AD are these belonging to and innervated by the cholinergic forebrain projection. It is a neuromodulatory system related to high cognitive functions. Deficits in these commands are the first clinical manifestations of AD [8].

Forebrain cholinergic neurons and areas extensively innervated by them (hippocampus and neocortex) contain the largest amounts of senile plaques. According to amyloid cascade hypothesis, widely accepted by scientists, senile plaques are primary factors causing neuronal death in AD, whereas neurofibrillary tangles are secondary [9]. The major constituent of senile plaques are peptides called β-amyloids, products of enzymatic cleavage of APP. Formation of senile plaques is a result of many years of accumulation of β-amyloids and other peptides forming extracellular insoluble aggregates. However it appears that in shorter periods APP and its metabolites demonstrate neuroprotective activity. The increased deposition of APP is a relatively quick reaction to factors deteriorating brain functioning, see for example:[10]. Direct neuroprotective effects were shown in the rat hippocampus [11]. They also were reported to protect cognitive functions following application of anticholinergic agent [12]. Recently Koudinov and Berezov have reviewed evidence showing a positive role of β-amyloids in the brain [13].

Cholinergic neurons display particular vulnerability to any negative factors affecting brain function, see for example: [14-16]. Ageing is a major process causing chronic deterioration of brain functioning, the cholinergic system being particularly susceptible and inducing long lasting overproduction of β-amyloids. If this process is aggravated by genetic and/or epigenetic factors it may eventually lead to development of AD.

The importance of cholesterol in the brain functioning is suggestively reflected by the fact that the human brain making up only 2% of total body weight contains as much as 25% of the total pool of this lipid [17]. To a big extent it is concentrated in myelin sheath. However there are also considerable amounts also in neuronal plasmalemma and in lipid rafts in particular. Lipid rafts seem to play a key role in transmembrane signalling processes, including synaptic transmission [18]. Importantly, APP is suggested to occur in lipid rafts [19,20].

The hypothesis aims at explaining this positive role of cholesterol in the brain in association with geriatric cholinergic deficits and APP metabolism.

## Presentation of the hypothesis

As suggested above age-related dysfunction in the forebrain cholinergic system results in APP hypermetabolism. However the mechanisms by which APP metabolites fulfil their neuroprotective functions despite some attempts, have not been fully elucidated. I hypothesise that these properties are due to the involvement of APP metabolites in the process of internalization of lipoproteins labelled with apoE, enriched with cholesterol. What could be the aim of this process ? There are data indicating a positive dependence of cholinergic receptors (both muscarinic and nicotinic) on cholesterol content in plasma membranes [21] and the mechanism of molecular interactions between cholesterol and nicotinic receptors have been proposed [22]. In contrast, receptors for monoaminergic agonists, seem to be negatively modulated by high membrane cholesterol [21,23]. In this respect increased uptake of cholesterol might be at least in part a process aiming at compensating cholinergic deficits and/or cholesterol deficits in AD caused for instance by genetic factors. Within the framework of this concept it is possible to explain the causal relationship between AD and a phenotype of apoE. The importance of apoE containing lipoproteins in neuroregenerative processes in connection to the role of cholesterol seems to be well established [20,24], although the role of APP and relationship of apoE-dependent transport with cholinergic transmission has not been fully clarified, despite some interesting proposals [24], which may be regarded as complementary to this hypothesis. There are three allele: ε2, ε3 and ε4 coding different isoforms of the protein. Expression of apoE4 isoform increases the risk of both sporadic and familial late onset of AD. Consequently one can assume that a higher demand of neurons for cholesterol results in higher production of β-amyloids which are engaged in internalization of apoE containing lipoproteins. Possibly interaction between the apoE4 isoform and β-amyloids in contrast to other isoforms is more prone to accumulation of insoluble aggregates and in addition might be less effective in cholesterol uptake as suggested in[20]. The relationship between levels of expression of APP metabolites, amount of apoE and cholesterol levels has been shown in several instances. An interesting example of such a relationship is provided by Howland et al [25] who carried out experiments exploring a mouse model of AD. In these mice exposure to a high cholesterol diet resulted in reduction of APP metabolites and concomitant increase of apoE. Similarly, it was reported that cells in culture exposed to high cholesterol reduced APP metabolism [26]. Reduction in APP metabolite production in conditions of high cholesterol in these publications may be explained by the negative feedback principle.

## Testing the hypothesis

The best way to test the hypothesis might be by experiments combining all of its main constituents. For instance by inducing APP hypermetabolism, and then verify whether supplementation of high cholesterol would result in:

- lowering of APP hypermetabolism

- better survival of neurons in crucial areas like the forebrain cholinergic system, hippocampus and some neocortical areas

- better preservation of cognitive functions

## Implications of the hypothesis

If the hypothesis is proved to be true it should first of all change negative attitude towards blood high cholesterol levels in clinical practice. In particular the use of statins in older subjects with neurological disorders should be revised. Recently there is an increasing number of reports indicating uncertainties related to this issue [20,27]. However this should be handled with care, since some authors even acknowledging the positive role of cholesterol in the brain do not exclude some beneficial actions of this group of drugs see e.g. [7]. Nevertheless, if the hypothesis is validated it may result in changes in some diet recommendations, especially while considering that definitely not in all cases high blood cholesterol must result in arteriosclerosis, this is supported by findings indicating homocysteine and not cholesterol as the primary vessel damaging factor in this disease [28], see also a strong criticism of the concept "blaming" cholesterol as a primary factor in the disease [29].

Moreover it would open up new alleys in brain function studies in general and AD in particular. It would imply the need of widening our understanding of the activity of cholesterol in the plasmalemma of neurons and mechanisms by which cholinergic receptors interact with plasmalemmal cholesterol.
